# Whole-Genome Sequencing of *Peribacillus frigoritolerans* Strain d21.2 Isolated in the Republic of Dagestan, Russia

**DOI:** 10.3390/microorganisms12122410

**Published:** 2024-11-24

**Authors:** Maria N. Romanenko, Anton E. Shikov, Iuliia A. Savina, Anton A. Nizhnikov, Kirill S. Antonets

**Affiliations:** 1 All-Russia Research Institute for Agricultural Microbiology, 196608 St. Petersburg, Russia; m.romanenko@arriam.ru (M.N.R.); a.shikov@arriam.ru (A.E.S.);; 2 Faculty of Biology, St. Petersburg State University, 199034 St. Petersburg, Russia

**Keywords:** *Peribacillus frigoritolerans*, draft genome, Illumina sequencing, mobile genetic elements, biosynthetic gene clusters, bactericidal activity, fungicidal properties

## Abstract

Pesticide-free agriculture is a fundamental pillar of environmentally friendly agriculture. To this end, there is an active search for new bacterial strains capable of synthesizing secondary metabolites and toxins that protect crops from pathogens and pests. In this study, we isolated a novel strain d21.2 of *Peribacillus frigoritolerans* from a soil sample collected in the Republic of Dagestan, Russia. Leveraging several bioinformatic approaches on Illumina-based whole-genome assembly, we revealed that the strain harbors certain insecticidal loci (coding for putative homologs of Bmp and Vpa) and also contains multiple BGCs (biosynthetic gene clusters), including paeninodin, koranimine, schizokinen, and fengycin. In total, 21 BGCs were predicted as synthesizing metabolites with bactericidal and/or fungicidal effects. Importantly, by applying a re-scaffolding pipeline, we managed to robustly predict MGEs (mobile genetic elements) associated with BGCs, implying high genetic plasticity. In addition, the d21.2’s genome was free from genes encoding for enteric toxins, implying its safety in use. A comparison with available genomes of the *Peribacillus frigoritolerans* strain revealed that the strain described here contains more functionally important loci than other members of the species. Therefore, strain d21.2 holds potential for use in agriculture due to the probable manifestation of bactericidal, fungicidal, growth-stimulating, and other useful properties. The assembled genome is available in the NCBI GeneBank under ASM4106054v1.

## 1. Introduction

*Peribacillus frigoritolerans* is a rod-shaped, Gram-positive bacterium belonging to the Bacillaceae family. Initially classified under the genus *Brevibacterium* as *Brevibacterium frigoritolerans*, it was later reclassified into the *Peribacillus* genus [1,2]. As the available data suggest, *P. frigoritolerans* could exhibit certain insecticidal properties. For instance, Selvakumar et al. (2011) [3] demonstrated that the *P. frigoritolerans* strain HSB-15 possessed entomopathogenic activity toward several representatives of Scarabaeidae, Coleoptera. In addition, various PGP (plant growth-promoting) *P. frigoritolerans* strains were reported [2]. Given a wide arsenal of bioactive compounds produced by the bacterium, other agriculturally important features, including fungicidal and bactericidal activities as well as the potential of the species as a biofertilizer and the agent for bioremediation, were described [4,5,6].

*P. frigoritolerans* was first isolated in 1967 from soil in Morocco [7]. However, its habitat is not restricted to soil only. Diverse strains were reported to reside in arctic snow [8], food products [9], contaminated anthropogenic zones [10,11], animals [12], and plants [4,5,13]. It is known for its ability to neutralize phosphates, which is viewed as a promising agent for ameliorating territories extensively treated with chemical pesticides such as phorate [6]. Nevertheless, the ecological role of the species remains poorly studied so far, albeit its appearance in the rhizosphere [13,14,15] and as an endophytic bacterium [16,17] coupled with PGP properties [13,16] suggest its primary role as a plant-associated microorganism whose presence in the plant microbiome is beneficial to hosts due to assistance in the uptake of nutritional and mineral substances [14,15,16] as well as protection from various phytopathogens [3,5,13,18].

Despite their promising applicability potential, bacteria of this genus are less frequently utilized for biopreparation production compared to the members of the *Bacillus* genus. Conducting an in-depth analysis, including genomic studies, will provide greater insight into the metabolic capacities of *P. frigoritolerans*. This approach will help evaluate their viability as biopreparation producers and the range of secondary metabolites they produce to ease strains’ selection for potential applications in biotechnology and agriculture. Such research could expand their role in sustainable farming practices.

Several works related to genome sequencing of *P. frigoritolerans* strains have been published [2,4,8,19,20,21,22,23]. However, it should be noted that these research items were primarily focused on general genomic features, leaving aside mining for functionally important genes such as BGCs (biosynthetic gene clusters) and insecticidal loci, and the description of such genomic traits remains scarce and constitutes a minor fraction of the present research [2,4], despite the amount of genomes currently available in the databases. For this reason, here, we performed an in-depth genomic survey of the strain d21.2 isolated in the Republic of Dagestan and conducted a comparative bioinformatic analysis of available *P. frigoritolerans* genomes to characterize genomic determinants potentially associated with the bioactivities of the bacterium. By mining the genomic data, we characterized the spectrum of BGCs that the strain harbors. We found that certain BGCs are associated with MGEs (mobile genetic elements) pointing to an active process of genome-wise adaptation. We believe that the provided data will assist in further understanding of *P. frigoritolerans* biology and developing biopreparations containing the species.

## 2. Materials and Methods

### 2.1. Isolation of the Strain from a Soil Sample

**Figure 1:**
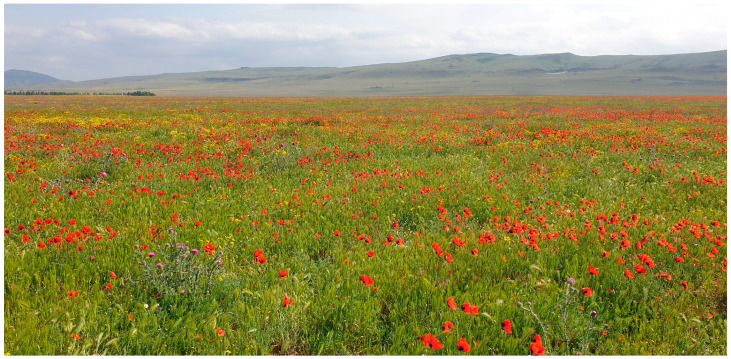
Soil sampling location.

The strain d21.2 was isolated from a soil sample collected in a poppy field (Figure 1) in the Republic of Dagestan, Russia (43.07264, 47.16567), in April 2022 and was stored in our laboratory collection in 25% glycerol solution at −80 °C.

**Figure 2:**
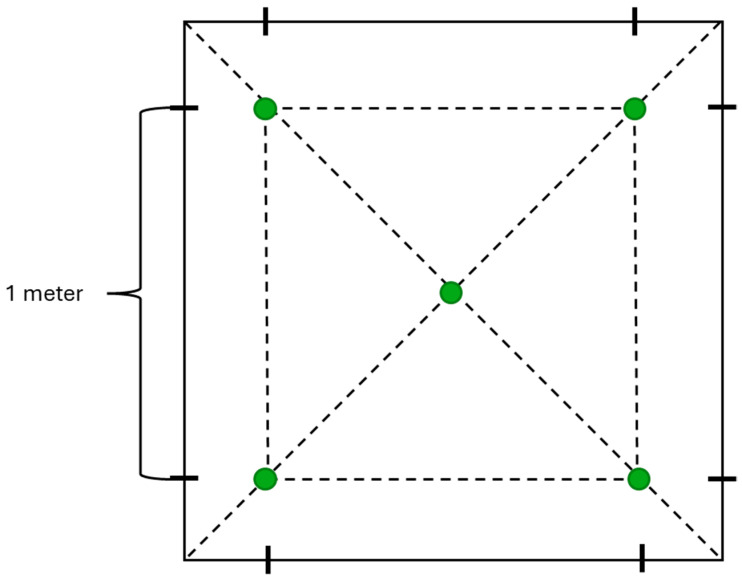
Protocol for sampling using the “envelope” method. Green circles indicate sampling areas.

The sampling followed this procedure: soil was collected from a depth of 3–5 cm using a sterile mini garden shovel and placed in a clean ziplock bag. Multiple samples were taken from the same location, approximately 1 m apart, using the “envelope” technique [24] (Figure 2).

To isolate the strain, 0.2 g of soil was suspended in 2 mL of sterile water and mixed thoroughly for 10 min to ensure full homogenization. This prepared solution was distributed into Eppendorf tubes (1 mL each) and heated at 80 °C for 30 min to eliminate non-spore-forming organisms and vegetative rod-shaped cells. Next, three serial dilutions (10×, 100×, and 1000×) were prepared, and 200 μL of each was spread onto Petri dishes containing selective T3 agar medium (tryptone 3 g/L; tryptose 2 g/L; yeast extract 1.5 g/L; NaH_2_PO_4_ × H_2_O 6.9 g/L; MnCl_2_ × 4H_2_O 0.008 g/L; agar 15 g/L; pH 6.8) [25] to promote spore formation. The plates were incubated at 28 °C for 72 h. The resulting colonies were then repeatedly transferred to fresh T3 agar plates until the culture was cleared of contaminants.

### 2.2. Morphological Description of the Strain

The vegetative cells were stained with Coomassie Brilliant Blue and examined under a light microscope (Carl Zeiss Axio Imager 2, Carl Zeiss Microscopy GmbH, Jena, Germany) to identify the morphology. Bacteria were cultured on CCY [26] agar for two days to induce sporulation. A small sample was suspended in sterile water, dried, stained for 1–15 min, rinsed with distilled water, and observed at 1000× magnification. Colony morphology was analyzed by growing bacteria on LB Miller agar (tryptone 10 g/L; yeast extract 5 g/L; sodium chloride 10 g/L; VWR International Ltd., Poole, UK) at 28 °C for one day, and features, like surface texture, color, profile, and edge shape, were recorded.

### 2.3. DNA Extraction, Quality Control, and Whole-Genome Sequencing

Total genomic DNA was extracted and qualitatively and quantitatively assessed according to the protocol described by Romanenko et al. (2023), with slight modifications at the DNA purification step [27]. After 12 h of incubation in Spizizen liquid medium [28,29], the bacterial culture was centrifuged and washed 3 times with EDTA/NaCl buffer. The cell pellet was resuspended in the above buffer and treated with Ribonuclease A. Cell lysis was initiated by the addition of lysozyme and mutanolysin from *Streptomyces globisporus* with further incubation at +37 °C for 60 min. To purify the sample from proteins, serial treatments with proteinase K, 10% sodium dodecyl sulfate, and the commercial Protein Precipitation Solution (Qiagen, Venlo, The Netherlands) were performed. For the latter, 200 µL of the solution was added to the sample, gently mixed, and incubated on ice for 60 min. Finally, the genomic DNA was precipitated with isopropanol, washed with 70% ethanol, and dissolved in Tris-EDTA buffer. The final concentration of the DNA was measured with a Qubit 3.0 fluorimeter using the Qubit dsDNA BR Assay kit (Thermo Fisher Scientific Inc., Eugene, OR, USA). Absorbance measurements of 260 nm/280 nm and 260 nm/230 nm were obtained with a ClarioSTAR Plus Multi-Mode Microplate Reader (BMG LABTECH, Ortenberg, Germany). Additional analysis of DNA samples was accomplished by 1% agarose gel electrophoresis.

### 2.4. Whole-Genome Sequencing, De Novo Genome Assembly and Annotation

Whole-genome sequencing was performed on the Illumina NovoSeq X platform (Illumina Inc., San Diego, CA, USA) in paired-end mode with a read length of 2 × 100 bp by Novogene Co., Ltd. (Beijing, China). The details of the annotation process are given in our previous research [27]. Briefly, we processed and quality-checked raw reads with FastQC v0.12.1 [30] and fastp v0.23.2 [31]. The reads were then used to produce genome assembly with SPAdes v3.15.4 [32] with subsequent quality assessment using QUAST v5.2.0 [33], BUSCO v5.4.2 [34], and CheckM v1.2.2 [35]. To obtain gene annotations, we picked the 10 closest genome assemblies according to ANI (Average Nucleotide Identity) values evaluated by fastANI v1.33 [36]. The subset was used for building a gene prediction model when running the Prokka v1.14.6 program [37]. We also used all protein sequences from reference BUSCO proteomes belonging to the Bacillales order as an annotation database.

At the next step, the assembled genome was screened for sequences of genes encoding various insecticidal toxins, as well as determinants of bactericidal, fungicidal, and other properties using BtToxin_Digger v1.0.10 [38], CryProcessor v1.0 [39], DeepBGC v0.1.30 [40], and antiSMASH v7.1.1 [41]. To assess the safety of the strain, we studied the output of the Btyper3 v3.4.0 [42] tool.

### 2.5. Scaffolding Genome Assembly and Detecting MGEs

We applied the RagTag v2.1.0 [43] utility implementing “correct”, “scaffold”, and “patch” procedures using the closest complete-level genome (GCF_030122925.1) as a reference. We then re-ran the mentioned annotation pipeline accordingly. Several tools were used for mining MGEs in both initial and corrected genome versions. To predict genomic islands, we utilized IslandPath-DIMOB v1.0.1_b [44] and SigiHMM v4.0 [45], prophages—DBSCAN-SWA v1.0 [46] and PhiSpy v3.7.8 [47], while insertion sequences were detected with ISEScan v1.7.2.3 [48]. To visualize genomic tracks and the loci-wise genomic properties of the assemblies, we applied CGView v1.13 [49] software and two R v4.4.2 packages, namely, ggplot2 v3.5.1 [50] and VennDiagram v1.7.3 [51].

### 2.6. Comparative Genome Analysis Procedure

To explore the genetic determinants of the representatives from the *P. frigoritolerans* species, we chose 50 genomes with the highest ANI estimates relative to our strain using a dataset of all genomes from the Bacillales order from the NCBI RefSeq database [52]. We then re-annotated them and revealed BGCs as well as toxin-encoding loci, as described above. To obtain core gene-based phylogeny, we reconstructed the pangenome including these references and the genome of strain d21.2 using Panaroo v1.2.8 [53], specifying the MAFFT v7.525 [54] aligner for performing gene-wise alignments. Core SNPs (single-nucleotide polymorphisms) were retrieved with SNP-sites v2.5.1 [55], and the resulting tree was built utilizing the FastTree v2.1.11 [56] software.

## 3. Results

### 3.1. Isolation and Characterization of the Strain According to Morphology

**Figure 3:**
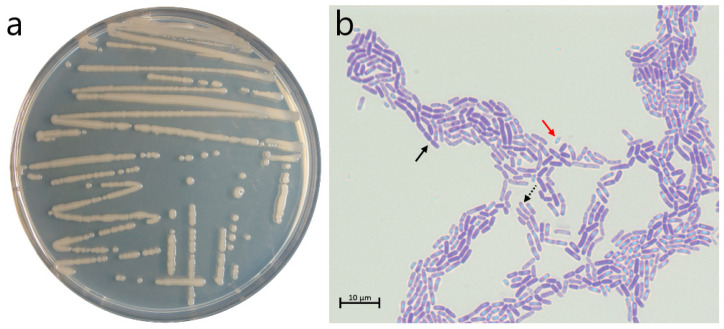
The morphology of the d21.2 strain colonies after 24 h of cultivation on LB medium (**a**) and (**b**) transition from vegetative to sporulating culture after 48 h of cultivation on CCY medium stained with Coomassie brilliant blue (1000× magnification, scale bar corresponding 10 µm). The black solid arrow shows the vegetative cell, the black dotted arrow—the spore inside the cell, and the red solid arrow—the spore.

The strain d21.2 was chosen from six representatives of the soil sample collected in the Republic of Dagestan. After 24 h of cultivation on the LB (Luria-Bertani, Miller, VWR International Ltd., Solon, OH, USA) agar nutrient medium, the strain formed circular, flat, yellowish-white colonies with entire margins and a glistening smooth surface (Figure 3a). On the second day of cultivation on the CCY [26] medium, the transition to the sporulation stage was observed. The bacterium formed rod-shaped vegetative cells collected in short chains with oval spores in the sub-terminal position (Figure 3b).

### 3.2. Analysis of Whole-Genome Sequencing (WGS) Results

Using paired reads with trimmed adapter sequences and the SPAdes v3.15.4 [32] software, we generated a draft genome assembly comprising 48 contigs with a total length of 5,638,879 base pairs and a GC content of 40.22%. The average sequencing coverage across contigs was 479. The assembly’s completeness level reached 98.91% coupled with a low contamination rate (1.82%). Additional genome metrics are provided in Table 1.

The examination with the BUSCO [34] program revealed that at least 99.8% of single-copy orthologues were fully assembled based on comparisons with the Bacillales_odb10 and Bacilli_odb10 databases (Table 2). These results further confirm the high quality and completeness of the genome assembly.

We then selected the 10 closest assemblies showing the highest similarity in nucleotide sequences to the genome of the studied strain based on ANI (Average Nucleotide Identity) estimates found with fastANI v1.33 [36] (Table 3). The close references were assigned to *P. frigoritolerans*, hence confirming the taxonomic attribution of strain d21.2 to this species.

After annotation with Prokka [37], we predicted 5426 coding sequences, only 700 of which coded for uncharacterized hypothetical proteins. According to the inferences obtained with BtToxin_Digger [38] and CryProcessor [39] utilities, the genome does not contain cry genes. However, the former tools reported two loci coding for the insecticidal toxin Vpa (recently known as Vip2), as well as the metalloproteinase Bmp1. Although Vpa toxins require the pair Vpb to enable activity [57], the similarity with known moieties is rather low (26%), implying that the predicted insecticidal activity of the strain requires further screening to identify potential novel types of toxins.

We then surveyed the strain’s metabolic capabilities by predicting BGCs with two programs with different underlying algorithms. The antiSMASH [41] tool identified nine biosynthetic gene clusters, listed in Table 4. The most notable sequence similarity was observed with known BGCs corresponding to paeninodin (100%) and koranimine (87%). Significant matches were also found with BGCs of schizokinen (60%) and fengycin (46.67%). Additionally, the DeepBGC [40] utility identified 21 gene clusters, of which 2 are responsible for the synthesis of secondary metabolites with both bactericidal and fungicidal properties, while the remaining have only bactericidal properties (Table S1). According to the output of Btyper3 [42], the strain is free from anthrax, emetic, cytotoxic, and diarrheal toxins. The absence of these detrimental virulence factors suggests the strain’s safety.

### 3.3. Connections Between MGEs and Functional Loci in Raw and Refined Genome Assembly

We then went on to characterize MGEs in the studied genome. It is generally accepted that fragmented multi-contig assemblies might be not suitable for mining MGEs [58]. Nevertheless, due to the significance of these predictions for understanding the dynamics of genomic architecture, we performed such an analysis with a preliminary refinement of the contigs by choosing the closest genome of a complete level as a reference for further sequence reconfiguration with the RagTag [43] tool. The most suitable genome was the assembly of the strain CF29 (GCF_030122925.1) comprising a single chromosomal sequence.

**Figure 4:**
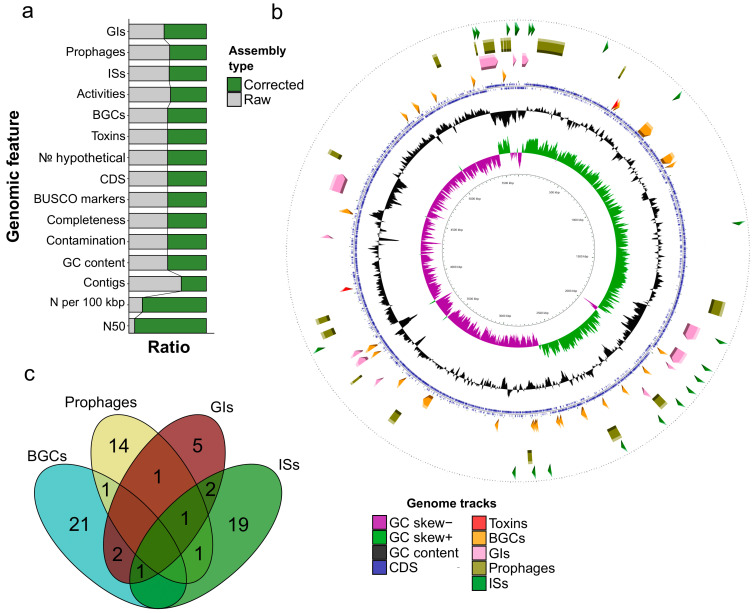
Overall and feature-wise genomic characterization of *P. frigoritolerans* d21.2’s assembly corrected according to the closest reference (GCF_030122925.1). (**a**). Comparison between raw and refined assemblies in terms of basic properties (number of contigs and CDS, quality metrics, etc.), and the abundance of certain loci, i.e., genes coding for insecticidal toxins, and BGCs as well as MGEs, namely, prophages, ISs, and GIs. The colored parts of the bar plots represent the ratio between the numeric values of the plotted feature. The exact figures are provided in Table S2. (**b**). The whole genome map of the corrected assembly. The inner circles demonstrate the GC distribution, while the outer tracks show the position of distinct loci colorized according to their types. (**c**). Relationships between the locations of BGCs and MGEs on the refined genome. The regions are considered overlapped in case the respective coordinates of compared loci intersect. The inspected coordinates are present in Table S3.

After the reconfiguration, the most drastic alterations occurred in reducing the number of contigs to 23 alongside a dramatic expansion of the N50 value to 5,402,018. At the same time, the proportion of ambiguous nucleotides increased substantially (Figure 4a, Table S2). The quality metrics remained untouched, whilst several notable changes affected the identification of MGEs. The corrected assembly bore more GIs (genomic islands) but contained fewer elements of other types, i.e., ISs (insertion sequences) and prophages. As can be inferred from the genomic tracks, the refinement procedure substantially leveled the GC distribution among the contigs and resulted in the lengthening of the predicted regions (Figure 4b and Figure S1a). While the RagTag-based procedure provides a certain beneficial effect on the assembly’s quality, we believe that it depends on the used references; thus, one should upload raw genomes to publicly available databases so that one can refine them when more phylogenetically close assemblies become available.

Having obtained a corrected genome, we proceeded with revealing connections between MGEs and certain loci to elucidate the genomic plasticity in terms of agriculturally valuable traits. To this end, we inspected whether the studied regions overlap (Table S3). We found no associations between insecticidal genes and MGEs of any kind, while BGCs fell into distinct MGEs, especially with GIs (Figure 4c). The same pattern was observed with the raw assembly as well (Figure S1b). Therefore, we showed that strain d21.2 possesses certain metabolic clusters that fall into mobile genomic blocks, likely allowing the strain to adjust its metabolism and occupy new ecological niches.

### 3.4. Comparative Genomic Analysis with P. frigoritolerans Strains

After obtaining a high-quality genome assembly of the *P. frigoritolerans* strain d21.2, we proceeded with a comprehensive genomic analysis, including 50 representatives of the species, to reveal unique features of the isolate described here. The metadata show that most of the strains were isolated from soil or rhizosphere (Table S4), while, in the context of the country of origin (if present), those isolated from the USA and China prevailed. Notably, four strains including ours were attributed to Russia (Table S4). After screening the genomes with BTyper3 [42], we revealed that all of the strains, including ours, are free from well-known enteric and anthrax-like toxins, namely, genes encoding for cereulide synthetase (*cesABCD*), non-hemolytic enterotoxins (*nheABC*), hemolysins (*hblABCD*), cytotoxin K (*cytK*), and sphingomyelinase (*sph*).

**Figure 5:**
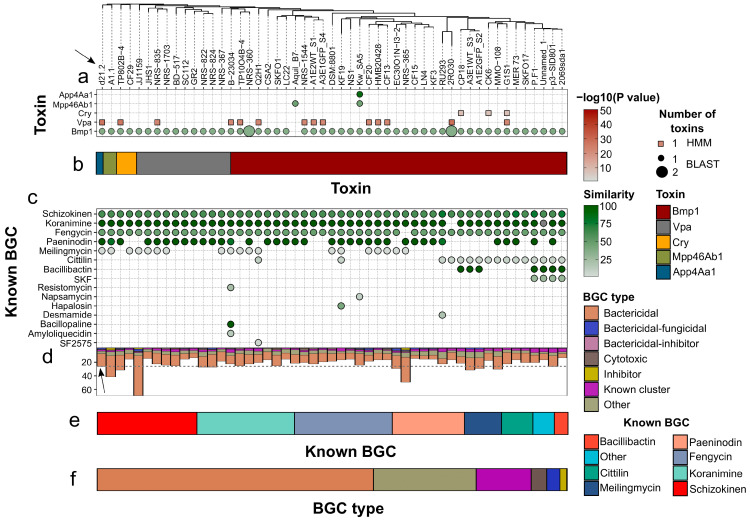
Comparative genomic analysis of the strain d21.2 with the closest 50 genomes of *P. frigoritolerans* isolates exhibiting the highest ANI values in terms of insecticidal loci and clusters responsible for the synthesis of secondary metabolites. (**a**) The composition of toxins predicted to be produced by the analyzed strains. The columns are organized according to the phylogeny reconstructed on the sequences of concatenated core genes. The adjacent tree is placed above. The size of the dots is proportional to the number of paralogs of a certain moiety. The shape indicates the method that detected a toxin sequence, namely, HMM (Hidden Markov Models) and BLAST with the color corresponding to the E-value and the similarity to the known homolog, respectively. (**b**) The total fraction of toxins identified in *P. frigoritolerans* genomes. The blocks are colorized according to the toxin group. (**c**) Known BGCs found in the genomic dataset. The order of columns corresponds to strains as in the toxins-based heatmap above. The circles are colorized according to the mean similarity with core genes of the known cluster. (**d**) The composition of BGCs detected with antiSMASH and DeepBGC in *P. frigoritolerans* strains. The color represents the classified BGC, i.e., predicted biological activity. If no activity was predicted, the BGC is marked as a known cluster (in case it exists) or other (lacking known hits and putative activities). (**e**) The frequency of known clusters found in the dataset. The color reflects the name of the product. (**f**). The overall distribution of BGCs present in *P. frigoritolerans* genomes. The blocks are colored according to the activity-wise classification mentioned above.

After mining loci encoding for pesticidal determinants, we found a total of five toxin types detected in the genomic dataset (Figure 5a, Table S5). More than 60% of genomes (32) were associated with one toxin hit only; thus, our strain contained more insecticidal loci compared to the average. Notably, homologs of Bmp1 were found in all isolates (Figure 5a) except for strain Aquil_B7, possessing loci encoding the Mpp46Ab1 homolog. Cry and Vpa loci were detected only by the HMM method, while Bmp1, Mpp46Ab1, and App4Aa1 were reported by the BLAST algorithm implemented in BtToxin_Digger, respectively. Since the CryProcessor tool did not identify the presence of Cry toxins possessing all three domains coupled with the absence of BLAST-based predictions, we could conclude that the analyzed strains do not produce these crystalline toxins. Regarding the overall distribution of toxin classes, we could conclude that Bmp1 and distant homologs of Vpa constitute a major fraction of insecticidal moieties found in 98% and 27.5% of isolates, respectively (Figure 5b, Table S6).

Next, we mined for BGCs in the analyzed genomes. We combined the outputs of the antiSMASH and DeepBGC programs to unify both known and putative metabolic clusters coupled with predicted activities (Table S7). We started with a distribution of known clusters and revealed that schizokinen was found in all genomes (Figure 5c). BGCSs related to koranimine and fengycin were identified in 50 genomes out of 51, while paeninodin, meilingmycin (the unknown analog with only 2% similarity), and cittilin were detected in 37, 19, and 16 strains (Table S8). Therefore, these six clusters constitute the highest fraction of *P. frigoritolerans* BGCs (Figure 5e) present in the genomic dataset analyzed here. The mean number of known BGCs in the genome constituted 4.7. Therefore, our strain was close to the average.

According to the distribution of chemical classes related to the predicted BGCs, almost a third (28.47%) of clusters were of unknown category, whereas the most frequently occurring known chemical groups were saccharides (17.58%), polyketides (11.45%), and terpenes (9.52%), with others constituting the remaining third (Table S9). We then classified all the BGCs detected by two programs according to the type and predicted activities (Figure 5d). On average, genomes possessed 24 BGCs, implying that, similar to pesticidal loci, our strain was associated with a higher number of metabolic clusters (28). In general, the vast majority of BGCs represented those producing metabolites with putative bactericidal activity (Table S10). Notably, almost 60% of the clusters were predicted to be related to bactericidal activities (Table S10, Figure 5f).

Altogether, the genomic analysis showed that the members of *P. frigoritolerans* with sequenced genomes hold agricultural potential due to the enrichment with bactericidal loci, and the absence of enteric toxins suggests the species could be applied as a biocontrol agent. In comparison to its relatives, the strain d21.2 was enriched with functionally important loci implying its prospectiveness.

## 4. Discussion

In this study, we performed a genomic analysis of *P. frigoritolerans* strain d21.2. As the species is close to the genus of *Bacillus*, especially the *Bacillus cereus sensu lato* group [59], a concern about the biosafety of the strain arises. Representatives of *B. cereus s.l.* often carry genes coding for enteric and anthrax toxins [60,61]. Since they are frequently isolated from food, surveillance of potentially pathogenic strains is needed [62]. This requirement is backed by the fact that some commercial biopreparations, including *P. frigoritolerans*, were reported to contain the *nheA* genes involved in the biosynthesis of non-hemolytic enterotoxins [63]. Therefore, we analyzed the composition of virulence factors and showed that strain d21.2 as well as its closest strains do not possess such pathogenic determinants, which highlights their biosafety.

One notable discovery of our study is the presence of homologs of known insecticidal loci, such as the one coding for Bmp1, presented in almost all genomes in the dataset (Figure 5a). The strain Kw SA5 contained App4Aa with high similarity (87.5%), implying its possible activity against *Plutella xylostella* [64]. Importantly, other loci, e.g., remote homologs of genes encoding for Cry and Vpa toxins with the latter identified in our strain, allow us to speculate that the species might possess certain insecticidal activities similar to what was described for strain HSB-15 [3]. Nevertheless, the genome of this strain has not been sequenced so far. Given that most of the identified pesticidal factors exhibit low similarity with the known ones (Table S5), one could expect novel insecticidal toxins produced by *P. frigoritolerans*, although unveiling their nature would require combined studies employing both NGS and experimental bioassays.

Using the bioinformatic analysis, we revealed that the strain is a predicted producer of a wide range of chemical substances (Table 4). One of them, paeninodin, is a lasso peptide falling into the RiPPs group (ribosomally synthesized and post-translationally modified peptides) [65]. Although it does not exhibit inhibitory effects on distinct bacterial species when used solely, it has been identified in bioactive PGP *Paenibacillus* strains [65]. This suggests that paeninodin could potentially contribute to activity when combined with other bactericidal and/or fungicidal metabolites.

Koranimine appears to hold great pesticidal potential. Montecillo and Bae (2022) [18] combined metabolomics and genomics approaches to investigate the chemical repertoire and biosynthetic potential of the bacterial endophyte *P. frigoritolerans* BE93, previously characterized as exhibiting nematicidal activity against *Bursaphelenchus xylophilus*. The analysis revealed that of all the moieties, the cyclic imine heptapeptide, koranimine, has potential nematicidal activity, thus highlighting the perspectives in developing nematocidal formulations based on *P. frigoritolerans* strains.

Fengycin is a cyclic lipopeptide known for its strong fungicidal and bactericidal activity. Fan et al. (2017) [66] showed that fengycin plays a major role in controlling apple ring rot disease. In a study by Medeot et al. (2020) [67], the anti-phytopathogenic effect of *Bacillus amyloliquefaciens* MEP218 was primarily based on cyclic lipopeptides (CLPs), particularly fengycins. The antibacterial activity of these fengycins was verified against *Xanthomonas axonopodis* pv. *vesicatoria* (Xav), which causes bacterial spot disease in plants, and *Pseudomonas aeruginosa* PA01, a human opportunistic pathogen.

Schizokinen, a citrate-containing dihydroxamate, is a siderophore produced by *Bacillus megaterium* and *Anabaena* sp. [68] Siderophores are low-molecular-weight, metal-chelating non-ribosomal peptides synthesized by some microorganisms and plants, especially under iron-deficient conditions [69]. It has been shown that the growth of *Bacillus megaferium* ATCC 19213 at extreme iron concentrations induced the secretion of the iron transport agent schizokinen [70]. The importance of siderophores is closely related to iron, which is an essential element for different biological processes [71]. Siderophores offer a wide range of beneficial functions. They can stimulate the growth of uncultured microorganisms [72,73,74] and enhance iron uptake in plants, boosting growth and yield [74,75,76]. These compounds serve as biocontrol agents, potentially replacing harmful pesticides by combating phytopathogens [74,77,78]. Siderophores also play a role in detoxifying heavy metal-contaminated environments, making them valuable for bioremediation [74,79,80,81]. Additionally, they act as biosensors to detect iron levels in various settings [74]. In medicine, they help deliver antibiotics to resistant bacteria using a “Trojan horse” mechanism, enhancing targeted treatments [82]. Thus, it can be assumed that schizokinene, being a siderophore, can potentially have a wide range of beneficial properties.

Having compared strain d21.2 with the closest genomes, we found that the composition of BGCs is, at some point, uniform with clusters controlling the production of schizokinen, koranimine, and fengycin being found in almost all 51 genomes (Figure 5c). Such a distribution might explain the observer inhibitory effects of *P. frigoritolerans* on various bacterial and fungal species, including phytopathogens. The bacterium was shown to exert an antagonistic effect on *Alternaria* spp. [2,5,21], *Botrytis cinerea* [2,17], *Fusarium* spp. [2,4,17,83], *Pythium* spp. [17,21], *Phytophthora* spp. [2], *Sclerotium rolfsii* [83], and *Rhizoctonia solani* [2,4,17]. Bactericidal properties are much less understood, albeit the existing observation made by Singh et al. (2015) [84] shows that silver nanoparticles produced by the strain strongly inhibit human pathogens, such as *Vibrio parahaemolyticus*, *Salmonella enterica*, and *Bacillus anthracis*, which implicates possible activity towards phytopathogens, which have not been tested yet. The anti-phytopathogenic potential of distinct strains varies substantially. For instance, strain BRN4, despite being entophyte in roots and shoots of the fennel plants, neither exhibited PGP properties nor affected *Fusarium* spp. and *Rhizoctonia solani* [85]. Strains HST10, HST17, and HRT8 residing in the rhizosphere of *Iris pseudacorus* plants were able to suppress *B. cinerea*, while HRT8 only showed significant activity against *Fusarium* spp. [17]. At the same time, HST17 inhibited the growth of *R. solani* while not affecting *Pythium ultimum*, while for strain HST10, the reverse was true [17].

To harness the reasons for the discrepancies mentioned above, it is advisable to apply bioinformatic analysis to reveal associations between genomic traits. While no genome sequences of the strains mentioned are available, we focused on the genomes included in our analysis. It is worth noting that the closest genome to d21.2 was strain A1.1, isolated from poultry farms in Belgorod, Russia (Table S4). Since the region is relatively close to the Republic of Dagestan, one could suppose the presence of bacterial populations with mutual gene flow. *P. frigoritolerans* A1.1 was inactive towards Gram-negative bacteria while exhibiting the effect on Gram-positive agents of human diseases and fungal phytopathogens, such as *Alternaria brassicicola*, *Pythium vexans*, and *Aspergillus unguis* [21], and possessed the same BGC composition as our strain, being associated, however, with more putative metabolic clusters in total (Figure 5c). The strain 2RO30 isolated from the canola rhizosphere was capable of suppressing a wide array of phytopathogens, namely, *Alternaria alternata*, *Phytophthora* spp., *B. cinerea*, and *Fusarium* species (*F. oxysporum*, *F. oxysporum*, and *F. oxysporum*) [2]. Świątczak et al. (2024) [2] reported that the strain contains the fengycin-producing BGC, while our analysis with preliminary re-annotation revealed another two clusters, namely, cittilin (Table S7). Notably, 2RO30 was low in the number of BGCs in comparison to the average estimates and was the only strain unable to produce koranimine according to genomic predictions (Figure 5c).

The current application of the species remains limited. Mostly, it is included in commercial biofertilizers as a supplement to other rhizobacteria, while commercial biopesticides, including the bacteria, are not described [63]. However, given the experimental validation for tested strains, we expect strain d21.2 described here to inhibit the growth of the pathogens described above, especially those affected by *P. frigoritolerans* A1.1 due to phylogenetic proximity and the same composition of BGCs. Altogether, the prevalence of metabolic clusters in strain d21.2 and its closest phylogenetic neighbors reflects the yet-unharnessed potential of the practical usage of the species in agriculture.

## 5. Conclusions

In summary, the analysis presented here describes the genetic architecture of the soil-dwelling *P. frigoritolerans* d21.2. Being safe in terms of virulence factors, enriched with BGCs of bactericidal, fungicidal, and putative nematocidal actions, and also possessing certain insecticidal loci, the strain deserves further testing. Moreover, the presence of multiple MGEs associated with metabolic clusters indicates probable horizontal gene transfer events; thus, the genome provided by us could help in untangling the evolutionary history of *P. frigoritolerans* by broadening the spectrum of available genomes. The loci present in the strains’ genome represent the markers of beneficial traits, and the fact that our strains possess them more than an average representative of the species, as shown by comparative genomic analysis, implies that the isolate described here could be a candidate for biopreparations as a potential biocontrol agent. The key findings from the genomic analysis are the abundance of multiple uncharacterized biosynthetic gene clusters, which could explain the unequal activities of the strains and the presence of insecticidal loci of low homology with known pesticidal moieties. Thus, our results emphasize that there is a need to carry out the screening for insecticidal, bactericidal, and fungicidal activities of the species coupled with genomic surveys to identify the genomic features responsible for agriculturally important characteristics to accelerate progress in developing biopreparations.

## Figures and Tables

**Table 1:** The main characteristics of the draft genome assembly of *P. frigoritolerans* strain d21.2.

Feature	Value
Total amount of contigs	48
Largest contig (number of nucleotides)	592,496
N50 value	432,533
N90 value	75,728
L50 value	6
L90 value	17
Number of properly paired reads (%)	99.06
Assembly completeness (%)	98.91
Suspected contamination (%)	1.82

**Table 2:** Estimation of the presence of BUSCO [34] markers in protein-coding genes presented in the assembly.

Database	Bacillales_odb10	Bacilli_odb10
Single-copy orthologues assembled completely	449 out of 450 (99.8%)	302 out of 302 (100.0%)
Orthologues present in one copy	444 out of 450 (98.7%)	301 out of 302 (99.7%)
Multi-copies orthologues	5 out of 450 (1.1%)	1 out of 302 (0.3%)
Fragmented sequences	0 out of 450 (0.0%)	0 out of 302 (0.0%)
Orthologues missing from the assembly	1 out of 450 (0.2%)	0 out of 302 (0.0%)
Total number of single-copy orthologues in the database	450	302

**Table 3:** The list of the phylogenetically closest assemblies relative to the genome of the studied strain according to the ANI value calculated with the fastANI v1.33 software.

NCBI RefSeq Assembly	Taxon	Strain	ANI
GCF_029625965	*P. frigoritolerans*	A1.1	98.78
GCF_007828935	*P. frigoritolerans*	VIVO01	98.66
GCF_030122925	*P. frigoritolerans*	CF29	98.56
GCF_026794285	*P. frigoritolerans*	TP802B-4	98.52
GCF_037027045	*P. frigoritolerans*	JJ1159	98.41
GCF_036220485	*P. frigoritolerans*	NRS-835	98.38
GCF_036219585	*P. frigoritolerans*	NRS-1703	98.33
GCF_026891895	*Peribacillus* sp.	AS_2	98.26
GCF_022394675	*P. frigoritolerans*	JHS1	98.25
GCF_036220565	*P. frigoritolerans*	NRS-822	98.18

**Table 4:** Selected biosynthetic gene clusters revealed in the analyzed genome. The similarity to the known clusters is calculated with the antiSMASH v7.1.1 program. The “–” symbol in the last three columns indicates the properties of the cluster were undefined. The total length of the regions is given in brackets after the genomic coordinates.

Contig	Region Product	Type	Number of CDS	Location (Relative Coordinate, b.p.)	Most Similar Known Cluster	Similarity, %
1	T3PKS ^1^	–	39	375,631–416,719 (total: 41,088)	–	–
	Terpene	–	21	551,614–572,432 (total: 20,818)	–	–
2	Terpene	–	18	189,404–211,299 (total: 21,895)	–	–
	NI-siderophore ^2^	Other ^5^	10	513,534–529,046 (total: 15,512)	Schizokinen	60
3	LAP ^3^	–	17	102,897–126,432 (total: 23,535)	–	–
5	Lassopeptide	RiPP ^6^	23	190,546–214,519 (total: 23,973)	Paeninodin	100
6	NRPS ^4^	Polyketide	38	63,960–108,027 (total: 44,067)	Meilingmycin	2
7	NRPS	NRP	46	237,872–298,196 (total: 60,324)	Koranimine	87
8	Betalactone	NRP	25	184,667–208,836 (total: 24,169)	Fengycin	46.67

^1^ Type III polyketide synthase; ^2^ NRPS-independent, IucA/IucC-like siderophores; ^3^ Linear azol(in)e-containing peptides; ^4^ Non-ribosomal peptide synthetase; ^5^ Cluster containing a secondary metabolite-related protein that does not fit into any other category; ^6^ Ribosomally synthesized and post-translationally modified peptide product.

## Data Availability

The raw genome sequencing data of Illumina HiSeq X were submitted to the NCBI SRA database in a FASTQ format with BioSample SAMN41858569, under BioProject PRJNA1124522. The assembled genome is available in the NCBI GeneBank under ASM4106054v1.

## Supplementary Materials

The following supporting information can be downloaded at: https://www.mdpi.com/article/10.3390/microorganisms12122410/s1, Figure S1: Genomic features of the raw SPAdes-assembled genome of the strain d21.2; Table S1: Biosynthetic gene clusters harbored in the genomic assembly predicted with the antiSMASH v7.1.1 and DeepBGC v0.1.30 programs; Table S2: Comparison between the initial and corrected assembly of the strain d21.2 regarding common genomic properties, functionally important loci, quality, and the presence of MGEs; Table S3: The summary of MGEs found in raw and RagTag-corrected genome sequences of the strain d21.2; Table S4: The metadata for the 50 closest *Peribacillus frigoritolerans* genomes according to pair-wise ANI values relative to our strain; Table S5: The distribution of toxins detected in the analyzed genomes using the BtToxin_Digger v1.0.10 software; Table S6: The overall frequency of insecticidal toxins identified in *Peribacillus frigoritolerans* genomes; Table S7: The list of BGCs detected in the studied genomic dataset; Table S8: The abundance of known BGCs among the studied genomes; Table S9: The percentage of predicted chemical classes in the overall sets of identified BGCs; Table S10: The frequency of BGC types according to their activities and annotations.

## References

[1] Montecillo J.A.V., Bae H. (2022). Reclassification of *Brevibacterium frigoritolerans* as *Peribacillus frigoritolerans* comb. nov. Based on Phylogenomics and Multiple Molecular Synapomorphies. Int. J. Syst. Evol. Microbiol..

[2] Świątczak J., Kalwasińska A., Brzezinska M.S. (2024). Plant Growth–Promoting Rhizobacteria: *Peribacillus frigoritolerans* 2RO30 and *Pseudomonas sivasensis* 2RO45 for Their Effect on Canola Growth under Controlled as Well as Natural Conditions. Front. Plant Sci..

[3] Selvakumar G., Sushil S.N., Stanley J., Mohan M., Deol A., Rai D., Ramkewal, Bhatt J.C., Gupta H.S. (2011). *Brevibacterium frigoritolerans* a Novel Entomopathogen of *Anomala dimidiata* and *Holotrichia longipennis* (Scarabaeidae: Coleoptera). Biocontrol Sci. Technol..

[4] Marik D., Sharma P., Chauhan N.S., Jangir N., Shekhawat R.S., Verma D., Mukherjee M., Abiala M., Roy C., Yadav P. (2024). *Peribacillus frigoritolerans* T7-IITJ, a Potential Biofertilizer, Induces Plant Growth-Promoting Genes of *Arabidopsis thaliana*. J. Appl. Microbiol..

[5] Chacón-López A., Guardado-Valdivia L., Bañuelos-González M., López-García U., Montalvo-González E., Arvizu-Gómez J., Stoll A., Aguilera S. (2021). Effect of Metabolites Produced by *Bacillus atrophaeus* and *Brevibacterium frigoritolerans* Strains on Postharvest Biocontrol of *Alternaria alternata* in Tomato (*Solanum lycopersicum* L.). Biocontrol Sci..

[6] Jariyal M., Gupta V.K., Mandal K., Jindal V. (2015). *Brevibacterium frigoritolerans* as a Novel Organism for the Bioremediation of Phorate. Bull. Environ. Contam. Toxicol..

[7] Delaporte B., Sasson A. (1967). [Study of Bacteria from Arid Soils of Morocco: *Brevibacterium haloterans* n. sp. and *Brevibacterium frigoritolerans* n. sp]. C. R. Acad. Hebd. Seances Acad. Sci. D.

[8] Choe Y.-H., Lee J.I., Kim M. (2022). Complete Genome Sequence of *Brevibacterium frigoritolerans* Ant232, Isolated from Antarctic Snow. Microbiol. Resour. Announc..

[9] Choi E.J., Lee S.H., Jung J.Y., Jeon C.O. (2013). *Brevibacterium jeotgali* sp. nov., Isolated from Jeotgal, a Traditional Korean Fermented Seafood. Int. J. Syst. Evol. Microbiol..

[10] Wufuer R., Li W., Wang S., Duo J. (2022). Isolation and Degradation Characteristics of PBAT Film Degrading Bacteria. Int. J. Environ. Res. Public Health.

[11] McLoon A.L., Awad T.T., Bogardus M.F., Buono M.G., Devine K.A., Draper R.M., Femenella B., Gallagher H.M., Morelock L.A., Razi M. (2022). Draft Genome Sequences for 6 Isolates of Endospore-Forming Class *Bacilli* Species Isolated from Soil from a Suburban, Wooded, Developed Space. Microbiol. Resour. Announc..

[12] Janakiev T., Milošević Đ., Petrović M., Miljković J., Stanković N., Zdravković D.S., Dimkić I. (2023). *Chironomus riparius* Larval Gut Bacteriobiota and Its Potential in Microplastic Degradation. Microb. Ecol..

[13] Lu P., Jiang K., Hao Y.-Q., Chu W.-Y., Xu Y.-D., Yang J.-Y., Chen J.-L., Zeng G.-H., Gu Z.-H., Zhao H.-X. (2021). Profiles of *Bacillus* Spp. Isolated from the Rhizosphere of *Suaeda glauca* and Their Potential to Promote Plant Growth and Suppress Fungal Phytopathogens. J. Microbiol. Biotechnol..

[14] Gupta A., Singh A.N., Tiwari R.K., Sahu P.K., Yadav J., Srivastava A.K., Kumar S. (2023). Salinity Alleviation and Reduction in Oxidative Stress by Endophytic and Rhizospheric Microbes in Two Rice Cultivars. Plants.

[15] Gupta A., Tiwari R.K., Shukla R., Singh A.N., Sahu P.K. (2023). Salinity Alleviator Bacteria in Rice (*Oryza sativa* L.), Their Colonization Efficacy, and Synergism with Melatonin. Front. Plant Sci..

[16] Jiang X., Li W.-W., Han M., Chen G., Wu J., Lai S., Fu Z., Zhang S., Deng W.-W., Gao L. (2022). Aluminum-Tolerant, Growth-Promoting Endophytic Bacteria as Contributors in Promoting Tea Plant Growth and Alleviating Aluminum Stress. Tree Physiol..

[17] Shurigin V., Alimov J., Davranov K., Gulyamova T., Egamberdieva D. (2022). The Diversity of Bacterial Endophytes from *Iris pseudacorus* L. and Their Plant Beneficial Traits. Curr. Res. Microb. Sci..

[18] Montecillo J.A.V., Bae H. (2022). In Silico Analysis of Koranimine, a Cyclic Imine Compound from *Peribacillus frigoritolerans* Reveals Potential Nematicidal Activity. Sci. Rep..

[19] Zhang C., Li X., Yin L., Liu C., Zou H., Wu Z., Zhang Z. (2019). Analysis of the Complete Genome Sequence of *Brevibacterium frigoritolerans* ZB201705 Isolated from Drought- and Salt-Stressed Rhizosphere Soil of Maize. Ann. Microbiol..

[20] Liu G.-H., Liu B., Wang J.-P., Che J.-M., Li P.-F. (2020). Reclassification of *Brevibacterium frigoritolerans* DSM 8801^T^ as *Bacillus frigoritolerans* Comb. Nov. Based on Genome Analysis. Curr. Microbiol..

[21] Senchenkov V.Y., Lyakhovchenko N.S., Nikishin I.A., Myagkov D.A., Chepurina A.A., Polivtseva V.N., Abashina T.N., Delegan Y.A., Nikulicheva T.B., Nikulin I.S. (2023). Whole-Genome Sequencing and Biotechnological Potential Assessment of Two Bacterial Strains Isolated from Poultry Farms in Belgorod, Russia. Microorganisms.

[22] González-Reguero D., Robas-Mora M., Alonso M.R., Fernández-Pastrana V.M., Lobo A.P., Gómez P.A.J. (2024). Induction of Phytoextraction, Phytoprotection and Growth Promotion Activities in *Lupinus albus* under Mercury Abiotic Stress Conditions by *Peribacillus frigoritolerans* subsp. *mercuritolerans* subsp. nov. Ecotoxicol. Environ. Saf..

[23] Jin M., Zhao Q., Zhou Z., Zhu L., Zhang Z., Jiang L. (2020). Draft Genome Sequence of a Potential Organic Phosphorus-Degrading Bacterium *Brevibacterium frigoritolerans* GD44, Isolated from Radioactive Soil in Xinjiang, China. Curr. Microbiol..

[24] Kapanadze K., Magalashvili A., Imnadze P. (2019). Distribution of Natural Radionuclides in the Soils and Assessment of Radiation Hazards in the Khrami Late Variscan Crystal Massif (Georgia). Heliyon.

[25] Travers R.S., Martin P.A.W., Reichelderfer C.F. (1987). Selective Process for Efficient Isolation of Soil *Bacillus* spp. Appl. Environ. Microbiol..

[26] Stewart G.S., Johnstone K., Hagelberg E., Ellar D.J. (1981). Commitment of Bacterial Spores to Germinate A Measure of the Trigger Reaction. Biochem. J..

[27] Romanenko M.N., Nesterenko M.A., Shikov A.E., Nizhnikov A.A., Antonets K.S. (2023). Draft Genome Sequence Data of *Lysinibacillus sphaericus* Strain 1795 with Insecticidal Properties. Data.

[28] Saleem F., Shakoori A. (2017). The First Cry2Ac-Type Protein Toxic to *Helicoverpa armigera*: Cloning and Overexpression of *cry2ac7* Gene from SBS-BT1 Strain of *Bacillus thuringiensis*. Toxins.

[29] Spizizen J. (1958). Transformation of Biochemically Deficient Strains of *Bacillus subtilis* by Deoxyribonucleate. Proc. Natl. Acad. Sci. USA.

[30] Andrews S. FastQC: A Quality Control Tool for High Throughput Sequence Data 2010. https://www.bioinformatics.babraham.ac.uk/projects/fastqc/.

[31] Chen S., Zhou Y., Chen Y., Gu J. (2018). Fastp: An Ultra-Fast All-in-One FASTQ Preprocessor. Bioinformatics.

[32] Bankevich A., Nurk S., Antipov D., Gurevich A.A., Dvorkin M., Kulikov A.S., Lesin V.M., Nikolenko S.I., Pham S., Prjibelski A.D. (2012). SPAdes: A New Genome Assembly Algorithm and Its Applications to Single-Cell Sequencing. J. Comput. Biol..

[33] Gurevich A., Saveliev V., Vyahhi N., Tesler G. (2013). QUAST: Quality Assessment Tool for Genome Assemblies. Bioinformatics.

[34] Manni M., Berkeley M.R., Seppey M., Simão F.A., Zdobnov E.M. (2021). BUSCO Update: Novel and Streamlined Workflows along with Broader and Deeper Phylogenetic Coverage for Scoring of Eukaryotic, Prokaryotic, and Viral Genomes. Mol. Biol. Evol..

[35] Parks D.H., Imelfort M., Skennerton C.T., Hugenholtz P., Tyson G.W. (2015). CheckM: Assessing the Quality of Microbial Genomes Recovered from Isolates, Single Cells, and Metagenomes. Genome Res..

[36] Jain C., Rodriguez-R L.M., Phillippy A.M., Konstantinidis K.T., Aluru S. (2018). High Throughput ANI Analysis of 90K Prokaryotic Genomes Reveals Clear Species Boundaries. Nat. Commun..

[37] Seemann T. (2014). Prokka: Rapid Prokaryotic Genome Annotation. Bioinformatics.

[38] Liu H., Zheng J., Bo D., Yu Y., Ye W., Peng D., Sun M. (2021). BtToxin_Digger: A Comprehensive and High-Throughput Pipeline for Mining Toxin Protein Genes from *Bacillus thuringiensis*. Bioinformatics.

[39] Shikov A.E., Malovichko Y.V., Skitchenko R.K., Nizhnikov A.A., Antonets K.S. (2020). No More Tears: Mining Sequencing Data for Novel *Bt* Cry Toxins with CryProcessor. Toxins.

[40] Hannigan G.D., Prihoda D., Palicka A., Soukup J., Klempir O., Rampula L., Durcak J., Wurst M., Kotowski J., Chang D. (2019). A Deep Learning Genome-Mining Strategy for Biosynthetic Gene Cluster Prediction. Nucleic Acids Res..

[41] Blin K., Shaw S., Augustijn H.E., Reitz Z.L., Biermann F., Alanjary M., Fetter A., Terlouw B.R., Metcalf W.W., Helfrich E.J.N. (2023). AntiSMASH 7.0: New and Improved Predictions for Detection, Regulation, Chemical Structures and Visualisation. Nucleic Acids Res..

[42] Carroll L.M., Cheng R.A., Kovac J. (2020). No Assembly Required: Using BTyper3 to Assess the Congruency of a Proposed Taxonomic Framework for the *Bacillus cereus* Group with Historical Typing Methods. Front. Microbiol..

[43] Alonge M., Lebeigle L., Kirsche M., Jenike K., Ou S., Aganezov S., Wang X., Lippman Z.B., Schatz M.C., Soyk S. (2022). Automated Assembly Scaffolding Using RagTag Elevates a New Tomato System for High-Throughput Genome Editing. Genome Biol..

[44] Bertelli C., Brinkman F.S.L. (2018). Improved Genomic Island Predictions with IslandPath-DIMOB. Bioinformatics.

[45] Waack S., Keller O., Asper R., Brodag T., Damm C., Fricke W.F., Surovcik K., Meinicke P., Merkl R. (2006). Score-Based Prediction of Genomic Islands in Prokaryotic Genomes Using Hidden Markov Models. BMC Bioinform..

[46] Gan R., Zhou F., Si Y., Yang H., Chen C., Ren C., Wu J., Zhang F. (2022). DBSCAN-SWA: An Integrated Tool for Rapid Prophage Detection and Annotation. Front. Genet..

[47] Akhter S., Aziz R.K., Edwards R.A. (2012). PhiSpy: A Novel Algorithm for Finding Prophages in Bacterial Genomes That Combines Similarity- and Composition-Based Strategies. Nucleic Acids Res..

[48] Xie Z., Tang H. (2017). ISEScan: Automated Identification of Insertion Sequence Elements in Prokaryotic Genomes. Bioinformatics.

[49] Stothard P., Wishart D.S. (2005). Circular Genome Visualization and Exploration Using CGView. Bioinformatics.

[50] Wickham H. (2016). Ggplot2: Elegant Graphics for Data Analysis.

[51] Chen H., Boutros P.C. (2011). VennDiagram: A Package for the Generation of Highly-Customizable Venn and Euler Diagrams in R. BMC Bioinform..

[52] O’Leary N.A., Wright M.W., Brister J.R., Ciufo S., Haddad D., McVeigh R., Rajput B., Robbertse B., Smith-White B., Ako-Adjei D. (2016). Reference Sequence (RefSeq) Database at NCBI: Current Status, Taxonomic Expansion, and Functional Annotation. Nucleic Acids Res..

[53] Tonkin-Hill G., MacAlasdair N., Ruis C., Weimann A., Horesh G., Lees J.A., Gladstone R.A., Lo S., Beaudoin C., Floto R.A. (2020). Producing Polished Prokaryotic Pangenomes with the Panaroo Pipeline. Genome Biol..

[54] Katoh K., Standley D.M. (2013). MAFFT Multiple Sequence Alignment Software Version 7: Improvements in Performance and Usability. Mol. Biol. Evol..

[55] Page A.J., Taylor B., Delaney A.J., Soares J., Seemann T., Keane J.A., Harris S.R. (2016). SNP-Sites: Rapid Efficient Extraction of SNPs from Multi-FASTA Alignments. Microb. Genom..

[56] Price M.N., Dehal P.S., Arkin A.P. (2010). FastTree 2—Approximately Maximum-Likelihood Trees for Large Alignments. PLoS ONE.

[57] Syed T., Askari M., Meng Z., Li Y., Abid M., Wei Y., Guo S., Liang C., Zhang R. (2020). Current Insights on Vegetative Insecticidal Proteins (Vip) as Next Generation Pest Killers. Toxins.

[58] Ricker N., Qian H., Fulthorpe R.R. (2012). The Limitations of Draft Assemblies for Understanding Prokaryotic Adaptation and Evolution. Genomics.

[59] Patel S., Gupta R.S. (2020). A Phylogenomic and Comparative Genomic Framework for Resolving the Polyphyly of the Genus *Bacillus*: Proposal for Six New Genera of *Bacillus* Species, *Peribacillus* gen. nov., *Cytobacillus* gen. nov., *Mesobacillus* gen. nov., *Neobacillus* gen. nov., *Metabacillus* gen. nov. and *Alkalihalobacillus* gen. nov. Int. J. Syst. Evol. Microbiol..

[60] Hoffmaster A.R., Ravel J., Rasko D.A., Chapman G.D., Chute M.D., Marston C.K., De B.K., Sacchi C.T., Fitzgerald C., Mayer L.W. (2004). Identification of Anthrax Toxin Genes in a *Bacillus cereus* Associated with an Illness Resembling Inhalation Anthrax. Proc. Natl. Acad. Sci. USA.

[61] Sánchez-Chica J., Correa M.M., Aceves-Diez A.E., Castañeda-Sandoval L.M. (2021). Enterotoxin Gene Distribution and Genotypes of *Bacillus cereus sensu lato* Isolated from Cassava Starch. Toxins.

[62] Friesema I.H., Slegers-Fitz-James I.A., Wit B., Franz E. (2022). Surveillance and Characteristics of Food-Borne Outbreaks in the Netherlands, 2006 to 2019. Eurosurveillance.

[63] Bulgari D., Filisetti S., Montagna M., Gobbi E., Faoro F. (2022). Pathogenic Potential of Bacteria Isolated from Commercial Biostimulants. Arch. Microbiol..

[64] Sampson K.S., Tomso D.J., Agarwal S., McNulty B., Campbell C. (2013). Toxin Genes and Methods for Their Use. U.S. Patent.

[65] Zhu S., Hegemann J.D., Fage C.D., Zimmermann M., Xie X., Linne U., Marahiel M.A. (2016). Insights into the Unique Phosphorylation of the Lasso Peptide Paeninodin. J. Biol. Chem..

[66] Fan H., Ru J., Zhang Y., Wang Q., Li Y. (2017). Fengycin Produced by *Bacillus subtilis* 9407 Plays a Major Role in the Biocontrol of Apple Ring Rot Disease. Microbiol. Res..

[67] Medeot D.B., Fernandez M., Morales G.M., Jofré E. (2020). Fengycins from *Bacillus amyloliquefaciens* MEP218 Exhibit Antibacterial Activity by Producing Alterations on the Cell Surface of the Pathogens *Xanthomonas axonopodis* pv. *vesicatoria* and *Pseudomonas aeruginosa* PA01. Front. Microbiol..

[68] Plowman J.E., Loehr T.M., Goldman S.J., Sanders-Loehr J. (1984). Structure and Siderophore Activity of Ferric Schizokinen. J. Inorg. Biochem..

[69] Khan A., Singh P., Srivastava A. (2018). Synthesis, Nature and Utility of Universal Iron Chelator—Siderophore: A Review. Microbiol. Res..

[70] Mullis K.B., Pollack J.R., Neilands J.B. (1971). Structure of Schizokinen, An Iron-Transport Compound from *Bacillus megaterium*. Biochemistry.

[71] Crosa J.H., Walsh C.T. (2002). Genetics and Assembly Line Enzymology of Siderophore Biosynthesis in Bacteria. Microbiol. Mol. Biol. Rev..

[72] Kaeberlein T., Lewis K., Epstein S.S. (2002). Isolating “Uncultivable” Microorganisms in Pure Culture in a Simulated Natural Environment. Science.

[73] Lewis K., Epstein S., D’Onofrio A., Ling L.L. (2010). Uncultured Microorganisms as a Source of Secondary Metabolites. J. Antibiot..

[74] Saha M., Sarkar S., Sarkar B., Sharma B.K., Bhattacharjee S., Tribedi P. (2016). Microbial Siderophores and Their Potential Applications: A Review. Environ. Sci. Pollut. Res..

[75] Rungin S., Indananda C., Suttiviriya P., Kruasuwan W., Jaemsaeng R., Thamchaipenet A. (2012). Plant Growth Enhancing Effects by a Siderophore-Producing Endophytic Streptomycete Isolated from a Thai Jasmine Rice Plant (*Oryza sativa* L. Cv. KDML105). Antonie Leeuwenhoek.

[76] Masalha J., Kosegarten H., Elmaci Ö., Mengel K. (2000). The Central Role of Microbial Activity for Iron Acquisition in Maize and Sunflower. Biol. Fertil. Soils.

[77] Beneduzi A., Ambrosini A., Passaglia L.M.P. (2012). Plant Growth-Promoting Rhizobacteria (PGPR): Their Potential as Antagonists and Biocontrol Agents. Genet. Mol. Biol..

[78] Ahmed E., Holmström S.J.M. (2014). Siderophores in Environmental Research: Roles and Applications. Microb. Biotechnol..

[79] O’Brien S., Hodgson D.J., Buckling A. (2014). Social Evolution of Toxic Metal Bioremediation in *Pseudomonas aeruginosa*. Proc. R. Soc. B Biol. Sci..

[80] Rajkumar M., Ae N., Prasad M.N.V., Freitas H. (2010). Potential of Siderophore-Producing Bacteria for Improving Heavy Metal Phytoextraction. Trends Biotechnol..

[81] Nair A., Juwarkar A.A., Singh S.K. (2007). Production and Characterization of Siderophores and Its Application in Arsenic Removal from Contaminated Soil. Water Air Soil. Pollut..

[82] Möllmann U., Heinisch L., Bauernfeind A., Köhler T., Ankel-Fuchs D. (2009). Siderophores as Drug Delivery Agents: Application of the “Trojan Horse” Strategy. BioMetals.

[83] Rasool A., Imran Mir M., Zulfajri M., Hanafiah M.M., Azeem Unnisa S., Mahboob M. (2021). Plant Growth Promoting and Antifungal Asset of Indigenous Rhizobacteria Secluded from Saffron (*Crocus sativus* L.) Rhizosphere. Microb. Pathog..

[84] Singh P., Kim Y.J., Yang D.-C., Singh H., Wang C., Farh M.E.-A., Hwang K.H. (2015). Biosynthesis, Characterization, and Antimicrobial Applications of Silver Nanoparticles. Int. J. Nanomed..

[85] Shurigin V., Li L., Alaylar B., Egamberdieva D., Liu Y.-H., Li W.-J. (2024). Plant Beneficial Traits of Endophytic Bacteria Associated with Fennel (*Foeniculum vulgare* Mill.). AIMS Microbiol..

