# Supplementary material for: Whole-Genome Sequencing of Peribacillus frigoritolerans Strain d21.2 Isolated in the Republic of Dagestan, Russia
Source: Microorganisms. 2024 Nov 24;12(12):2410. doi: 10.3390/microorganisms12122410 (PMC11678259; doi:10.3390/microorganisms12122410)
Supplement: Supplementary file 1 [file microorganisms-12-02410-s001.zip › Supplementary_Tables_S1-S3_Figure_S1.pdf]

Supporting Information for

Whole genome sequencing of strain d21.2 isolated in the Republic of Dagestan

Maria N. Romanenko[1,2], Anton E. Shikov[1,2], Iuliia A. Savina[1], Anton A. Nizhnikov[1,2], Kirill S. Antonets[1,2,*]

[1]Laboratory for Proteomics of Supra-Organismal Systems, All-Russia Research Institute for Agricultural Microbiology (ARRIAM), 196608 St. Petersburg, Russia;

[2]Faculty of Biology, St. Petersburg State University (SPbSU), 199034 St. Petersburg, Russia.

* **Correspondence**: Kirill S. Antonets, k.antonets@arriam.ru

This file includes:

Figure S1

Tables S1-S3

Description of tables S1-S8

Supporting Figures

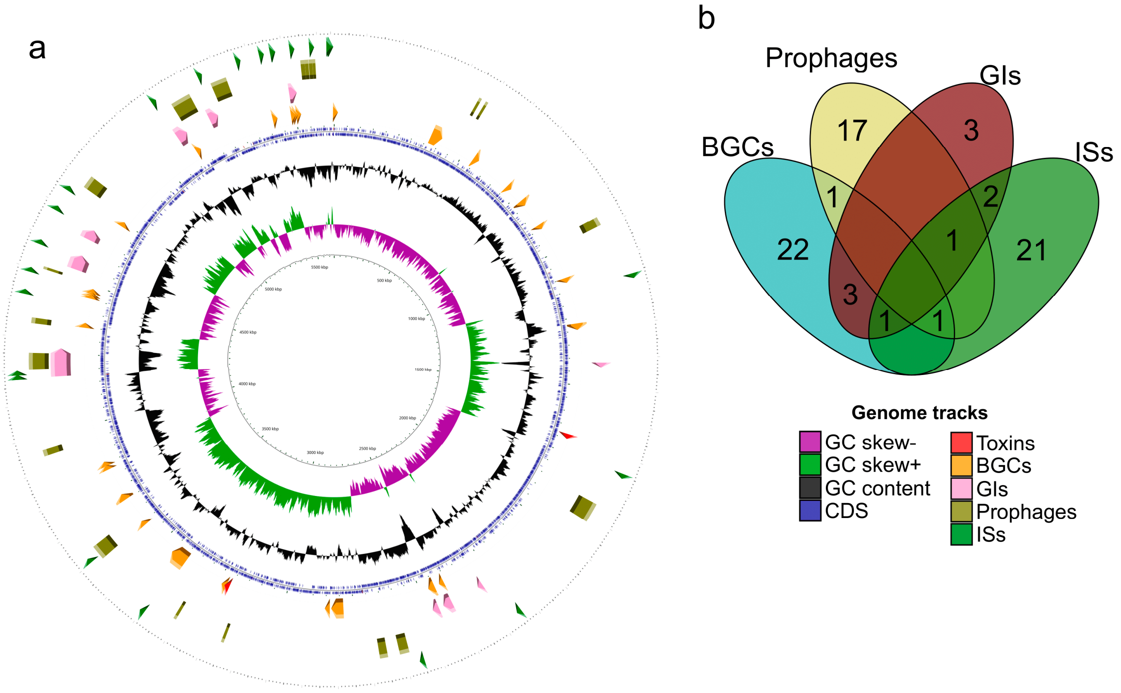

Figure S1. Genomic features of the raw SPAdes-assembled [1] genome of the strain d21.2. (**a**). The whole-genome map of the raw assembly. The inner circles show the distribution of the GC content (including skews and general average), while the outer circles depict selected loci of interest (toxin-encoding genes and BGCs) and MGEs of different kinds. The blocks on the map are colorized according to the type of the loci. (**b**). The number of overlapping genomic regions (BGCs) and various mobile genetic elements such as prophages, insertion sequences (ISs), and genomic islands (GIs). The regions are placed in the intersections if the coordinates of the respective loci overlap with each other. The used coordinate mappings are listed in Table S3.

Supporting Tables

Table S1. Biosynthetic gene clusters harbored in the genomic assembly predicted with the antiSMASH v7.1.1 [2] and DeepBGC v0.1.30 [3] programs. The score reflects the accuracy of cluster prediction obtained with the DeepBGC v0.1.30 program, while the similarity to the known clusters is calculated with the antiSMASH v7.1.1 program. The "–" symbol indicates that the biosynthetic cluster was found by only one program out of the two used.

Contig	Tool	Region product/Activity	Type/Class	Number of CDS within a biosynthetic cluster	Location (relative coordinate, b.p.)	Most similar known cluster	Similarity, %	Score
1	antiSMASH	T3PKS [1]	–	39	375 631 – 416 719 (total: 41 088)	–	–	–
		Terpene	–	21	551 614 – 572 432 (total: 20 818)	–	–	–
	DeepBGC	Antibacterial	Polyketide	–	15 369 – 17 440 (total: 2 071)	–	–	0.83542
2	antiSMASH	Terpene	–	18	189 404 – 211 299 (total: 21 895)	–	–	–
		NI-siderophore [2]	Other [5]	10	513 534 – 529 046 (total: 15 512)	Schizokinen	60	–
	DeepBGC	Antibacterial	Other	–	125 123 – 126 191 (total: 1 068)	–	–	0.79526
		Antibacterial	Saccharide	–	299 687 – 314 962 (total: 15 275)	–	–	0.84165
3	antiSMASH	LAP [3]	–	17	102 897 – 126 432 (total: 23 535)	–	–	–
5	antiSMASH/ DeepBGC	Lassopeptide/ Antibacterial	RiPP [6]/ Saccharide	23	190 546 – 222 749 (total: 32 203)	Paeninodin	100	0.97291
	DeepBGC	Antibacterial	–	–	163 098 – 163 311 (total: 213)	–		0.70458
6	antiSMASH/ DeepBGC	NRPS [4]/ Antibacterial	Polyketide/ NRP	38	63 960 – 108 027 (total: 44 067)	Meilingmycin	2	0.7799
	DeepBGC	Antibacterial	Polyketide- Terpene	–	121 474 –122 868 (total: 1 394)	–		0.81167
7	antiSMASH/ DeepBGC	NRPS/Antibacterial	NRP/ NRP	46	237 872 – 298 196 (total: 60 324)	Koranimine	87	0.8721
	DeepBGC	Antibacterial	–	–	75 827 – 76 57 (total: 750)	–	–	0.71152

8	antiSMASH	Betalactone	NRP	25	184 667 – 208 836 (total: 24 169)	Fengycin	46.67	–
	DeepBGC	Antibacterial-antifungal	Polyketide	–	74 691 – 81 075 (total: 6384)	–	–	0.71805
	DeepBGC	Antibacterial	–	–	316 853 – 319 813 (total: 2960)	–	–	0.77132
	DeepBGC	Antibacterial	Saccharide	–	324 918 – 339 393 (total: 14 475)	–	–	0.9813
10	DeepBGC	Antibacterial	–	–	180 132 – 182 871 (total: 2 739)	–	–	0.76629
11	DeepBGC	Antibacterial-antifungal	Polyketide	–	105 365 – 106 556 (total: 1 191)	–	–	0.7161
	DeepBGC	Antibacterial	–	–	112 817 – 113 822 (total: 1 005)	–	–	0.7184
	DeepBGC	Antibacterial	Saccharide	–	122 746 – 127 539 (total: 4 793)	–	–	0.82167
13	DeepBGC	Antibacterial	–	–	61 931 – 64 994 (total: 3 063)	–	–	0.72834
17	DeepBGC	Antibacterial	–	–	70 782 – 73 364 (total: 2 582)	–	–	0.75333
23	DeepBGC	Antibacterial	Polyketide	–	28 863 – 32 629 (total: 3 766)	–	–	0.82667
26	DeepBGC	Antibacterial	–	–	3 138 – 3 831 (total: 693)	–	–	0.73138
	DeepBGC	Antibacterial	RiPP	–	20 653 – 24 837 (total: 4 184)	–	–	0.80063

[1] Type III polyketide synthase;
[2] NRPS-independent, IucA/IucC-like siderophores;
[3] Linear azol(in)e-containing peptides;
[4] Non-ribosomal peptide synthetase;
[5] Cluster containing a secondary metabolite-related protein that does not fit into any other category;
[6] Ribosomally synthesised and post-translationally modified peptide product.

Table S2. Comparison between the initial and corrected assembly of the strain d21.2 regarding common genomic properties, functionally important loci, quality, and the presence of MGEs (Mobile Genetic Elements). The corrected assembly was obtained with the RagTag tool [4] using the closest genome (GCF_030122925.1) of the complete level. The content of the table is plotted in Figure 2a.

Assembly type	Genomic feature*	Value
Raw	Number of contigs	48
	N50	432,533
	N per 100 kbp	15.48
	GC content	40.22%
	Completeness	98.91%
	Contamination	1.82%
	Single-copy BUSCO markers (bacillales_odb10)	99.8%
	Number of CDS	5426
	Number of hypothetical proteins	700
	Number of toxins	2
	Number of BGCs (antiSMASH)	9
	Number of BGCs (deepBGC)	22
	Number of BGCs (total)	28
	Number of prophages	20
	Number of GIs	10
	Number of ISs	26
Corrected	Number of contigs	23
	N50	5,402,018
	N per 100 kbp	73.96
	GC content	40.22%
	Completeness	98.91%
	Contamination	1.82%
	Single-copy BUSCO markers (bacillales_odb10)	99.3%
	Number of CDS	5424
	Number of hypothetical proteins	702
	Number of toxins	2
	Number of BGCs (antiSMASH)	9
	Number of BGCs (deepBGC)	19
	Number of BGCs (total)	25
	Number of prophages	18
	Number of GIs	12
	Number of ISs	24

* CDS – Coding sequence, BGCs – Biosynthetic Gene Clusters, GIs – Genomic Islands, ISs – insertion sequences.

Table S3. The summary of MGEs found in raw and RagTag-corrected [4] genome sequences of the strain d21.2. Listed are genomic loci coding for insecticidal toxins and BGCs as well as MGEs, namely, prophages, ISs, and GIs. The provided data includes coordinates of the loci (contig as well as relative starting and ending positions), the tool that reported the moiety, and the metadata (if present). In case the region was detected by multiple instruments, a concatenated name is given.

Loci_type	Contig	Start	Stop	Tool	Metadata	Assembly type
Toxins	scf00000001	468613	470766	BtToxinDigger	Vpa	corr
Toxins	scf00000001	3974151	3975068	BtToxinDigger	Bmp1-other	corr
BGCs	scf00000001	643514	703315	antismash_deepbgc	koranimine	corr
BGCs	scf00000001	836278	880345	antismash_deepbgc	meilingmycin	corr
BGCs	scf00000001	3636987	3660960	antismash_deepbgc	paeninodin	corr
BGCs	scf00000001	2405963	2430132	antiSMASH	fengycin	corr
BGCs	scf00000001	3229476	3270564	antiSMASH	T3PKS	corr

BGCs	scf00000001	3406156	3426213	antiSMASH	terpene	corr
BGCs	scf00000001	4364794	4388329	antiSMASH	LAP	corr
BGCs	scf00000001	5021377	5043272	antiSMASH	terpene	corr
BGCs	scf00000001	5345507	5361019	antiSMASH	schizokinen	corr
BGCs	scf00000001	481036	481786	DeepBGC	antibacterial	corr
BGCs	scf00000001	893792	895186	DeepBGC	antibacterial	corr
BGCs	scf00000001	1902580	1903837	DeepBGC	antibacterial	corr
BGCs	scf00000001	2028101	2031164	DeepBGC	antibacterial	corr
BGCs	scf00000001	2176748	2178464	DeepBGC	antibacterial	corr
BGCs	scf00000001	2295987	2302371	DeepBGC	antibacterial-antifungal	corr
BGCs	scf00000001	2538149	2541109	DeepBGC	antibacterial	corr
BGCs	scf00000001	2546214	2564023	DeepBGC	antibacterial	corr
BGCs	scf00000001	2666156	2667347	DeepBGC	antibacterial-antifungal	corr
BGCs	scf00000001	2673608	2674613	DeepBGC	antibacterial	corr
BGCs	scf00000001	2683537	2688330	DeepBGC	antibacterial	corr
BGCs	scf00000001	2869214	2871285	DeepBGC	antibacterial	corr
BGCs	scf00000001	3609539	3609752	DeepBGC	antibacterial	corr
BGCs	scf00000001	4957096	4958164	DeepBGC	antibacterial	corr
BGCs	scf00000001	5131660	5146935	DeepBGC	antibacterial	corr
BGCs	seq00000003	3497	8327	DeepBGC	antibacterial	corr
Prophages	seq00000009	34	8675	PhiSpy	pp20	corr
Prophages	seq00000007	122	10800	PhiSpy	pp18	corr
Prophages	seq00000000	29728	76748	Phispy_DBSCAN-SWA	Staphylococcus_phage(33.33%)	corr
Prophages	seq00000008	348	11262	PhiSpy	pp19	corr
Prophages	seq00000003	396	9785	PhiSpy	pp17	corr
Prophages	scf00000001	23395	138689	PhiSpy	pp1	corr
Prophages	scf00000001	1568508	1632156	PhiSpy	pp2	corr
Prophages	scf00000001	2064055	2100668	PhiSpy	pp3	corr
Prophages	scf00000001	2303011	2346251	PhiSpy	pp4	corr
Prophages	scf00000001	2698768	2717233	PhiSpy	pp5	corr
Prophages	scf00000001	3316435	3347592	PhiSpy	pp6	corr
Prophages	scf00000001	3559845	3569856	PhiSpy	pp7	corr
Prophages	scf00000001	3785082	3815806	PhiSpy	pp8	corr
Prophages	scf00000001	3851260	3876487	PhiSpy	pp9	corr
Prophages	scf00000001	4595499	4617730	PhiSpy	pp12	corr
Prophages	scf00000001	5208890	5229090	PhiSpy	pp13	corr
Prophages	scf00000001	5393302	5401072	PhiSpy	pp14	corr
Prophages	scf00000001	407956	416247	DBSCAN-SWA	Synechococcus_phage(50.0%)	corr
GIs	seq00000012	245	3497	sigiHMM	sigiHMM_GI_7	corr
GIs	seq00000000	602	86334	IslandPath_sigiHMM	sigiHMM_GI_6	corr
GIs	scf00000000	19232	50353	islandpath	gi1	corr
GIs	scf00000001	1710948	1783789	IslandPath_sigiHMM	sigiHMM_GI_1	corr
GIs	scf00000001	4453212	4542154	IslandPath_sigiHMM	sigiHMM_GI_4	corr
GIs	scf00000001	1842077	1910510	islandpath	gi3	corr
GIs	scf00000001	1929624	1943862	islandpath	gi4	corr
GIs	scf00000001	2096109	2115379	islandpath	gi5	corr
GIs	scf00000001	3489765	3506725	islandpath	gi6	corr

GIs	scf00000001	3607017	3637012	islandpath	gi7	corr
GIs	scf00000001	3653845	3675541	sigiHMM	sigiHMM_GI_2	corr
GIs	scf00000001	4232761	4244412	sigiHMM	sigiHMM_GI_3	corr
ISs	scf00000000	49384	50597	ISscan	new	corr
ISs	scf00000001	34	1412	ISscan	IS21	corr
ISs	scf00000001	306338	308044	ISscan	IS110	corr
ISs	scf00000001	661060	661347	ISscan	IS3	corr
ISs	scf00000001	1238498	1239336	ISscan	IS3	corr
ISs	scf00000001	1731371	1732439	ISscan	IS110	corr
ISs	scf00000001	1853775	1855016	ISscan	IS3	corr
ISs	scf00000001	1907880	1909118	ISscan	IS1182	corr
ISs	scf00000001	1964185	1965866	ISscan	IS1182	corr
ISs	scf00000001	2031451	2031839	ISscan	IS3	corr
ISs	scf00000001	2132325	2133707	ISscan	IS110	corr
ISs	scf00000001	2318265	2319659	ISscan	IS1182	corr
ISs	scf00000001	2631844	2632637	ISscan	IS1182	corr
ISs	scf00000001	2696386	2697155	ISscan	IS5	corr
ISs	scf00000001	2776188	2777140	ISscan	IS3	corr
ISs	scf00000001	3757846	3758082	ISscan	IS1182	corr
ISs	seq00000000	3457	5101	ISscan	IS4	corr
ISs	seq00000000	16359	17223	ISscan	IS110	corr
ISs	seq00000009	110	901	ISscan	IS110	corr
ISs	seq00000016	100	1454	ISscan	IS110	corr
ISs	seq00000019	50	609	ISscan	IS110	corr
ISs	seq00000020	40	680	ISscan	IS110	corr
ISs	seq00000021	58	600	ISscan	IS3	corr
ISs	seq00000022	32	268	ISscan	IS110	corr
Toxins	NODE_3	516158	517075	BtToxinDigger	Bmp1-other	raw
Toxins	NODE_7	63404	65557	BtToxinDigger	Vpa	raw
BGCs	NODE_13	61931	64994	DeepBGC	antibacterial	raw
BGCs	NODE_5	190546	214519	antismash_deepbgc	paeninodin	raw
BGCs	NODE_5	163098	163311	DeepBGC	antibacterial	raw
BGCs	NODE_8	184667	208836	antiSMASH	fengycin	raw
BGCs	NODE_8	74691	81075	DeepBGC	antibacterial-antifungal	raw
BGCs	NODE_8	316853	319813	DeepBGC	antibacterial	raw
BGCs	NODE_8	324918	339393	DeepBGC	antibacterial	raw
BGCs	NODE_3	102897	126432	antiSMASH	LAP	raw
BGCs	NODE_26	3138	3831	DeepBGC	antibacterial	raw
BGCs	NODE_26	5242	16433	DeepBGC	cytotoxic	raw
BGCs	NODE_26	20653	24837	DeepBGC	antibacterial	raw
BGCs	NODE_17	70782	73364	DeepBGC	antibacterial	raw
BGCs	NODE_23	28863	32629	DeepBGC	antibacterial	raw
BGCs	NODE_6	63960	108027	antismash_deepbgc	meilingmycin	raw
BGCs	NODE_6	121474	122868	DeepBGC	antibacterial	raw
BGCs	NODE_1	375631	416719	antiSMASH	T3PKS	raw
BGCs	NODE_1	551614	572432	antiSMASH	terpene	raw
BGCs	NODE_1	15369	17440	DeepBGC	antibacterial	raw
BGCs	NODE_10	180132	182871	DeepBGC	antibacterial	raw
BGCs	NODE_11	105365	106556	DeepBGC	antibacterial-antifungal	raw
BGCs	NODE_11	112817	113822	DeepBGC	antibacterial	raw

BGCs	NODE_11	122746	127539	DeepBGC	antibacterial	raw
BGCs	NODE_2	189404	211299	antiSMASH	terpene	raw
BGCs	NODE_2	513534	529046	antiSMASH	schizokinen	raw
BGCs	NODE_2	125123	126191	DeepBGC	antibacterial	raw
BGCs	NODE_2	299687	314962	DeepBGC	antibacterial	raw
BGCs	NODE_7	237872	298196	antismash_deepbgc	koranimine	raw
BGCs	NODE_7	75827	76577	DeepBGC	antibacterial	raw
Prophages	NODE_13	93271	131656	PhiSpy	pp12	raw
Prophages	NODE_20	8054	56735	PhiSpy	pp14	raw
Prophages	NODE_5	340122	368947	PhiSpy	pp5	raw
Prophages	NODE_5	405163	429470	PhiSpy	pp6	raw
Prophages	NODE_28	298	12880	PhiSpy	pp15	raw
Prophages	NODE_29	122	10800	PhiSpy	pp16	raw
Prophages	NODE_8	76983	121782	PhiSpy	pp7	raw
Prophages	NODE_4	105707	165153	PhiSpy	pp3	raw
Prophages	NODE_30	348	11262	PhiSpy	pp17	raw
Prophages	NODE_1	483738	491688	PhiSpy	pp1	raw
Prophages	NODE_1	461506	469726	DBSCAN-SWA	Ostreococcus_lucimarinus_virus(16.67%)	raw
Prophages	NODE_10	26960	76748	Phispy_DBSCAN-SWA	Staphylococcus_phage(33.33%)	raw
Prophages	NODE_10	165797	182871	PhiSpy	pp10	raw
Prophages	NODE_11	148782	156442	PhiSpy	pp11	raw
Prophages	NODE_2	372343	398864	PhiSpy	pp2	raw
Prophages	NODE_18	401	61082	PhiSpy	pp13	raw
Prophages	NODE_9	88543	107699	PhiSpy	pp8	raw
Prophages	NODE_31	34	8675	PhiSpy	pp18	raw
Prophages	NODE_7	2747	11038	DBSCAN-SWA	Synechococcus_phage(50.0%)	raw
Prophages	NODE_7	160056	168612	DBSCAN-SWA	Staphylococcus_phage(42.86%)	raw
GIs	NODE_5	43324	60284	islandpath	gi1	raw
GIs	NODE_5	160576	190571	islandpath	gi2	raw
GIs	NODE_5	207404	229100	sigiHMM	sigiHMM_GI_2	raw
GIs	NODE_26	256	25464	IslandPath_sigiHMM	sigiHMM_GI_6	raw
GIs	NODE_3	246814	258465	sigiHMM	sigiHMM_GI_1	raw
GIs	NODE_17	35710	71202	IslandPath_sigiHMM	sigiHMM_GI_5	raw
GIs	NODE_12	233	50236	islandpath	gi4	raw
GIs	NODE_12	107500	142246	islandpath	gi5	raw
GIs	NODE_10	602	94244	IslandPath_sigiHMM	sigiHMM_GI_4	raw
GIs	NODE_19	19232	50353	islandpath	gi7	raw
ISs	NODE_10	3457	5101	ISscan	IS4	raw
ISs	NODE_10	16359	17223	ISscan	IS110	raw
ISs	NODE_11	71053	71846	ISscan	IS1182	raw
ISs	NODE_11	135595	136364	ISscan	IS5	raw
ISs	NODE_12	40158	41129	ISscan	IS3	raw
ISs	NODE_12	148642	148881	ISscan	IS110	raw

ISs	NODE_13	65281	65669	ISscan	IS3	raw
ISs	NODE_17	37277	38515	ISscan	IS1182	raw
ISs	NODE_19	49384	50597	ISscan	new	raw
ISs	NODE_2	569678	571430	ISscan	IS21	raw
ISs	NODE_21	49264	50209	ISscan	IS110	raw
ISs	NODE_23	33601	33803	ISscan	IS3	raw
ISs	NODE_24	31536	32039	ISscan	IS3	raw
ISs	NODE_25	205	1886	ISscan	IS1182	raw
ISs	NODE_27	137	541	ISscan	IS110	raw
ISs	NODE_31	110	901	ISscan	IS110	raw
ISs	NODE_4	61	310	ISscan	IS110	raw
ISs	NODE_4	492481	493314	ISscan	IS3	raw
ISs	NODE_41	100	1454	ISscan	IS110	raw
ISs	NODE_44	50	609	ISscan	IS110	raw
ISs	NODE_45	40	680	ISscan	IS110	raw

Description of Supplementary Tables

Table S1. Biosynthetic gene clusters harbored in the genomic assembly predicted with the antiSMASH v7.1.1 and DeepBGC v0.1.30 programs. The score reflects the accuracy of cluster prediction obtained with the DeepBGC v0.1.30 program, while the similarity to the known clusters is calculated with the antiSMASH v7.1.1 program. The "–" symbol indicates that the biosynthetic cluster was found by only one program out of the two used.

Table S2. Comparison between the initial and corrected assembly of the strain d21.2 regarding common genomic properties, functionally important loci, quality, and the presence of MGEs (Mobile Genetic Elements). The corrected assembly was obtained with the RagTag tool using the closest genome (GCF_030122925.1) of the complete level. The content of the table is plotted in Figure 2a.

Table S3. The summary of MGEs found in raw and RagTag-corrected genome sequences of the strain d21.2. Listed are genomic loci coding for insecticidal toxins and BGCs as well as MGEs, namely, prophages, ISs, and GIs. The provided data includes coordinates of the loci (contig as well as relative starting and ending positions), the tool that reported the moiety, and the metadata (if present). In case the region was detected by multiple instruments, a concatenated name is given.

Table S4. The metadata for the 50 closest *Peribacillus frigoritolerans* genomes according to pair-wise ANI values relative to our strain. Listed are the accessions in the NCBI RefSeq database, the organism and the strain attributed to the genome as well as the country of origin and isolation source.

Table S5. The distribution of toxins detected in the analyzed genomes using the BtToxin_Digger v1.0.10 software. For each toxin found in the strain ("*Tox*") the underlying detection algorithm, namely HMM (Hidden Markov Model) and BLAST is specified ("*Type*"). The E-values ("*HMM E-value*") and the identity ("*Identity*") with the closest known homolog are given for these methods, respectively.

Table S6. The overall frequency of insecticidal toxins identified in *Peribacillus frigoritolerans* genomes.

Table S7. The list of BGCs (Biosynthetic Gene Clusters) detected in the studied genomic dataset. The overall predictions by antiSMASH v7.1.1 and DeepBGC v0.1.30 programs are presented. In case the BGC was detected by multiple instruments, a concatenated name is given. Presented are, the coordinates of the regions, the number of CDS (Coding Sequences), the chemical class, the name of the most similar known cluster coupled with mean sequence similarity, and predicted activity coupled with a score. In case, a certain feature was not detected by the programs, the "–" symbol is given.

Table S8. The abundance of known BGCs among the studied genomes.

Table S9. The percentage of predicted chemical classes in the overall sets of identified BGCs.

Table S10. The frequency of BGC types according to their activities and annotations. The classification is predominantly based on the predicted biological activity. If no activity was predicted, the BGC is marked as a known cluster (in case it exists) or other (lacking known hits and putative activities)

References

1. Bankevich, A.; Nurk, S.; Antipov, D.; Gurevich, A.A.; Dvorkin, M.; Kulikov, A.S.; Lesin, V.M.; Nikolenko, S.I.; Pham, S.; Prjibelski, A.D.; et al. SPAdes: A New Genome Assembly Algorithm and Its Applications to Single-Cell Sequencing. *Journal of Computational Biology* **2012**, *19*, 455–477, doi:10.1089/cmb.2012.0021.
2. Blin, K.; Shaw, S.; Augustijn, H.E.; Reitz, Z.L.; Biermann, F.; Alanjary, M.; Fetter, A.; Terlouw, B.R.; Metcalf, W.W.; Helfrich, E.J.N.; et al. AntiSMASH 7.0: New and Improved Predictions for Detection, Regulation, Chemical Structures and Visualisation. *Nucleic Acids Res* **2023**, *51*, W46–W50, doi:10.1093/nar/gkad344.
3. Hannigan, G.D.; Prihoda, D.; Palicka, A.; Soukup, J.; Klempir, O.; Rampula, L.; Durcak, J.; Wurst, M.; Kotowski, J.; Chang, D.; et al. A Deep Learning Genome-Mining Strategy for Biosynthetic Gene Cluster Prediction. *Nucleic Acids Res* **2019**, *47*, e110–e110, doi:10.1093/nar/gkz654.
4. Alonge, M.; Lebeigle, L.; Kirsche, M.; Jenike, K.; Ou, S.; Aganezov, S.; Wang, X.; Lippman, Z.B.; Schatz, M.C.; Soyk, S. Automated Assembly Scaffolding Using RagTag Elevates a New Tomato System for High-Throughput Genome Editing. *Genome Biol* **2022**, *23*, 258, doi:10.1186/s13059-022-02823-7.
